# New prognostic factors and scoring system for patients with skeletal metastasis

**DOI:** 10.1002/cam4.292

**Published:** 2014-07-10

**Authors:** Hirohisa Katagiri, Rieko Okada, Tatsuya Takagi, Mitsuru Takahashi, Hideki Murata, Hideyuki Harada, Tetsuo Nishimura, Hirofumi Asakura, Hirofumi Ogawa

**Affiliations:** 1 Division of Orthopaedic Oncology, Shizuoka Cancer Center HospitalNagaizumi, Shizuoka, Japan; 2 Department of Preventive Medicine, Nagoya University, Graduate School of MedicineNagoya, Japan; 3 Division of Radiation Oncology, Shizuoka Cancer Center HospitalNagaizumi, Shizuoka, Japan

**Keywords:** Laboratory data, multivariate analysis, prognostic factor, prognostic scoring system, skeletal metastasis

## Abstract

The aim of this study was to update a previous scoring system for patients with skeletal metastases, that was proposed by Katagiri et al. in 2005, by introducing a new factor (laboratory data) and analyzing a new patient cohort. Between January 2005 and January 2008, we treated 808 patients with symptomatic skeletal metastases. They were prospectively registered regardless of their treatments, and the last follow-up evaluation was performed in 2012. There were 441 male and 367 female patients with a median age of 64 years. Of these patients, 749 were treated nonsurgically while the remaining 59 underwent surgery for skeletal metastasis. A multivariate analysis was conducted using the Cox proportional hazards model. We identified six significant prognostic factors for survival, namely, the primary lesion, visceral or cerebral metastases, abnormal laboratory data, poor performance status, previous chemotherapy, and multiple skeletal metastases. The first three factors had a larger impact than the remaining three. The prognostic score was calculated by adding together all the scores for individual factors. With a prognostic score of ≥7, the survival rate was 27% at 6 months, and only 6% at 1 year. In contrast, patients with a prognostic score of ≤3 had a survival rate of 91% at 1 year, and 78% at 2 years. Comparing the revised system with the previous one, there was a significantly lower number of wrongly predicted patients using the revised system. This revised scoring system was able to predict the survival rates of patients with skeletal metastases more accurately than the previous system and may be useful for selecting an optimal treatment.

## Introduction

Accurate data regarding the life expectancy of patients with skeletal metastasis are necessary so that appropriate treatment recommendations can be made. Currently, radiotherapy is a main stream treatment, but some patients with fracture or spinal instability require surgery if their life expectancy is not short. Conversely, some patients with short life expectancy should receive radiotherapy or best supportive care alone, even though they have fractures or spinal cord compression.

Palliative radiotherapy for painful bone metastasis is well established. Recently, short-course radiotherapy has been found to have similar outcomes to long-course radiotherapy 1. However, for patients with relatively longer expected survival, a longer course of radiotherapy is recommended because in-field recurrence rates are higher for short-course radiotherapy 2,3.

Regarding the surgery of extremity bone metastasis, there are two options, namely, osteosynthesis or excision followed by prosthetic replacement. Osteosynthesis is associated with fewer complications but has the increased risk of implant fracture if the patients have longer survival times. Conversely, prosthetic replacement is preferred if favorable survival is expected 4,5. Similarly, there are two procedures for the treatment of spinal metastasis. One is posterior decompression followed by instrumentation, and the other is excisional surgery; they are selected in accordance with life expectancy 6,7.

Most previous studies regarding prognostic factors for patients with bone metastases have had some bias regarding patient selection, because they analyzed patients treated either exclusively with surgery or exclusively with radiotherapy 3,6,8–10. In 2005, Katagiri et al. 11. and Tokuhashi et al. 7. reported a scoring system involving the analysis of surgically as well as nonsurgically treated patients. However, there was a need to reconsider the impact of the primary tumor on survival, because the development of effective targeted and selected chemotherapeutic regimens in the treatment of advanced cancer might have a positive impact on survival. Furthermore, previous prognostic models have not taken laboratory data into consideration, although laboratory data are useful indicators of patient prognosis for some malignancies 3,6–9,11. This study was designed to update a previous scoring system for patients with skeletal metastases proposed by Katagiri et al. 11, by introducing a new factor involving laboratory data and analyzing a prospectively registered new patient cohort.

## Materials and Methods

Between January 2005 and January 2008, a total of 958 patients were prospectively registered at the time of detection of symptomatic bone metastasis. Among them, we excluded those who had already undergone treatment at other institutes, or had not been treated at our institution. Consequently, our study group comprised 808 consecutive patients who had undergone surgical and/or nonsurgical treatment, or palliative care for skeletal metastases at our institute. The patients were prospectively followed and the last follow-up evaluation was performed in January 2012. There were 441 male and 367 female patients with a median age of 64 (range, 8–94) years.

Of the 808 patients, 779 (96%) were followed up for a minimum of 24 months, unless death supervened, during which time 29 were lost to follow-up. These 29 patients were treated as “censored observations.” Two deaths from causes other than malignancy were also treated as censored observations. The median follow-up periods were 6.4 (range, 0.25–77) months for patients dying from malignant disease, and 53.9 (range, 1–82) months for survivors. Multiple myeloma requiring orthopedic care or radiotherapy was treated as a skeletal metastasis 3,5,8,12.

Lung carcinoma was the most common primary lesion (26%) in the patient population. Other lesions were carcinoma of the breast (17%), colon and rectum (9%), stomach (6%), prostate (5%), and liver (5%). The primary lesion was not found in 16 patients despite thorough investigation (Table 1).

**Table 1:** Type of primary tumor, patient median survival, and classification

Primary lesion	Number of patients	(%)	Median survival (months)	Group
Lung cancer				
NSC with molecularly targeted therapy^1^	54	6.7	15.2	M
Other lung cancer	156	19.3	4.8	R
Brest cancer				
Hormone independent	78	9.7	10.3	M
Hormone dependent	63	7.8	34.0	S
Colon and rectal cancer	72	8.9	4.4	R
Gastric cancer	47	5.8	3.6	R
Prostate cancer				
Hormone independent	24	3.0	15.0	M
Hormone dependent	18	2.2	32.0	S
Hepatocellular carcinoma	40	5.0	7.4	R
Pancreatic cancer	27	3.3	4.0	R
Head and neck cancer	25	3.1	2.3	R
Other urological cancer (urethral cancer, etc.)	24	3.0	3.8	R
Esophageal cancer	22	2.7	2.0	R
Renal cell carcinoma	22	2.7	11.8	M
Malignant lymphoma	17	2.1	51.7	S
Multiple myeloma	15	1.9	38.1	S
Thyroid cancer	13	1.6	46.7	S
Sarcoma	12	1.5	11.6	M
Malignant melanoma	11	1.4	2.1	R
Gallbladder cancer	9	1.1	7.4	R
Cervical cancer	9	1.1	3.1	R
Other gynecological cancer	10	1.2	14.5	M
Unknown origin	16	2.0	4.5	R
Others	24	3.0	11.8	M
Total	808	100	7.7	

S, slow growth group; M, moderate growth group; R, rapid growth group; NSC, non-small cell.

1 Molecularly targeted agents: gefitinib and/or erlotinib.

### Treatment

Treatment for the skeletal lesion, including the surgical indication and radiation planning, was decided on at the bone metastasis board meeting at our hospital. Of the patients, 749 (93%) were treated nonsurgically while the remaining 59 (7%) underwent surgery for skeletal metastasis. Of those treated without surgery, 67 (8%) were given palliative care alone and radiotherapy was performed in 623.

Of the 59 patients treated with surgery, 12 were operated on for spinal metastasis, 45 for extremity metastasis, and two for pelvic lesions. The surgical indication for spinal metastasis included the following: incomplete palsy with painful spinal instability; a localized lesion; radioresistant tumor; and an expected survival time ≥6 months. Surgery for extremity metastasis was performed in patients with an expected survival time of ≥2 months. All patients gave their informed consent for each treatment.

### Imaging study

The spread of skeletal metastases was determined by means of bone scanning using 99^m^ Tc methylene diphosphonate in 440 patients (54%), and positron emission tomography with ([18]F)fluoro-2-deoxy-d-glucose (FDG-PET) scanning in 118 (15%). In 242 patients (30%), magnetic resonance imaging (MRI) and/or computed tomography (CT) combined with plain X-ray imaging were used to determine the spread of bone metastases. Visceral metastases were assessed using enhanced CT or PET-CT. Brain metastasis was assessed by MRI or enhanced CT if necessary.

### Statistical analysis

Twelve potentially prognostic factors were investigated (Table 2). Each was grouped in up to three categories for statistical analysis. Patients were categorized into age groups, namely, ≤64 or ≥65 years (the median age of the entire cohort was 64 years). Performance status (PS) was evaluated using the Eastern Cooperative Oncology Group Performance Status (ECOG PS) scale and patients were categorized into two groups, namely, ECOG PS 0–2 and 3–4. Patient neurological deficit was divided into two groups, namely, the group where useful motor function had been restored (Frankel types D, E) and the group where it had not been restored (Frankel types A, B, C) 13.

**Table 2:** Distribution of potentially prognostic factors

Prognostic factors	Subgroups	Number of patients	(%)
Patient-related factor			
Gender	Female	367	45
	Male	441	55
Age (years)	≤64	416	51
	≥65	392	49
ECOG performance status	PS 0–2	580	72
	PS 3–4	228	28
Neurological deficits	Frankel D, E	765	86
	Frankel A, B, C	43	14
Laboratory data^1^	Normal	185	23
	Abnormal^2^	457	57
	Critical^3^	150	19
Primary-site-related factor			
Primary site	Slow growth	126	16
	Moderate growth	224	28
	Rapid growth	458	57
Visceral or brain metastasis^4^	No	121	15
	Nodular metastasis	428	53
	Disseminated metastasis	245	30
Remaining primary lesion	No	272	34
	Yes	536	66
Previous chemotherapy	No	354	44
	Yes	454	56
Skeletal-metastasis-related factor			
Location of skeletal metastasis	Axial bone	528	65
	Axial bone and proximal limb	257	32
	Spreading to distal limb	23	3
Multiple skeletal metastases	No	200	25
	Yes	608	75
Pathological fracture	No	545	68
	Yes	261	32

ECOG, Eastern Cooperative Oncology Group.

1 792 subjects.

2 Abnormal: CRP ≥ 0.4 mg/dL, LDH ≥ 250 IU/L, or serum albumin <3.7 g/dL.

3 Critical: platelet count <100,000/*μ*L, serum calcium level ≥10.3 mg/dL, or total bilirubin ≥1.4.

4 794 subjects.

Laboratory data at the time of bone metastasis detection were investigated as a potential prognostic factor. We selected six laboratory data parameters namely: C-reactive protein (CRP); lactate dehydrogenase (LDH); serum albumin; serum calcium corrected for albumin level; platelet count; and total bilirubin.

CRP and LDH have been reported to be prognostic factors for patients with some malignancies 14,15. Serum albumin level is accepted as an indicator of patient nutrition status as well as an indicator of the general health condition of patients with hepatocellular carcinoma 16. Serum bilirubin levels are not only regarded as an essential factor in the Child-Pugh classification for hepatocellular carcinoma but also often indicate serious problems in the liver or bile duct 16. Thrombocytopenia often suggests cancer dissemination to the bone marrow, and hypercalcemia can endanger patient lives. Those patients who showed no abnormalities in these data were categorized as normal. Elevated CRP (≥0.4 mg/dL), LDH (≥250 IU/L), or hypoalbuminemia (<3.7 g/dL) were categorized as abnormal because they do not directly threaten patient lives. In contrast, changes in serum calcium level, thrombocytopenia, and hyperbilirubinemia can directly threaten lives. Therefore, if a patient showed any of the following abnormalities, their data were categorized as critical: thrombocytopenia (<100,000/*μ*L); hypercalcemia (≥10.3 mg/dL); or hyperbilirubinemia (total bilirubin ≥1.4 mg/dL). The abnormal ranges of CRP, total bilirubin, and serum calcium were determined according to *Harrison's Principles of Internal Medicine* 17.

Primary cancer was classified into three groups, namely, tumors that exhibited rapid growth, moderate growth, or slow growth, according to the median survival period of patients with every malignancy in this cohort calculated using the Kaplan–Meier method. To determine the survival period, we divided lung cancer patients into two subgroups, namely, lung cancer patients who were indicated for treatment with molecularly targeted agents (gefitinib and/or erlotinib), and other lung cancer patients. These two subgroups of patients with lung cancer were treated as having different tumors when calculating their survival. In addition, prostate and breast cancer patients were divided according to their sensitivity to hormonal therapy. Patients who had already undergone treatment with multiple hormonal therapy agents, or breast cancer patients with neither estrogen nor progesterone receptors, were considered to be hormone independent.

Patients with cancers who had a median survival time of >20 months were classified into the slow growth group; this included multiple myeloma, malignant lymphoma, thyroid cancer, hormone-dependent prostate cancer, and hormone-dependent breast cancer. Patients with cancers with a median survival time from 10 to 20 months were classified into the moderate growth group, and those with a median survival time of <10 months were classified into the rapid growth group (Tables 1 and 2).

Visceral or cerebral metastases were grouped into three categories: no visceral or cerebral metastasis; ordinary nodular metastasis; and disseminated metastasis such as pleural, peritoneal, or leptomeningeal dissemination. The condition of the primary lesion was classified into two categories according to whether it was already cured, or was untreated and recurring. Previous chemotherapy was classified into two categories, namely, no previous chemotherapy and previous chemotherapy.

The sites of skeletal metastases were divided into three categories: limited to axial bone; limited to axial bone and proximal extremity bone; and with metastatic spread below the elbow or knee. The skeletal metastatic load was divided into two categories, solitary and multiple skeletal metastases. Pathological fractures were also divided into two categories on imaging, as being either present or not present (Table 2).

Rates of patient survival were calculated using the Kaplan–Meier method. First, all of the factors shown in Table 2 were included as explanatory variables in a Cox proportional hazards survival analysis. Results of the multivariate analyses were expressed in terms of a hazard ratio with 95% confidence intervals. The significance level was set at a two-sided 5%. Next, we fitted the Cox proportional hazards model to the data, including significant factors from the initial analysis. On the basis of the results, significant factors and hazard ratio were obtained. In this analysis, categorical variables with more than two modalities were recorded using dummy variables. We used STATA version 9 software (StataCorp., College Station, TX) for multivariate analysis.

## Results

### Survival rates and prognostic factors

**Figure 1:**
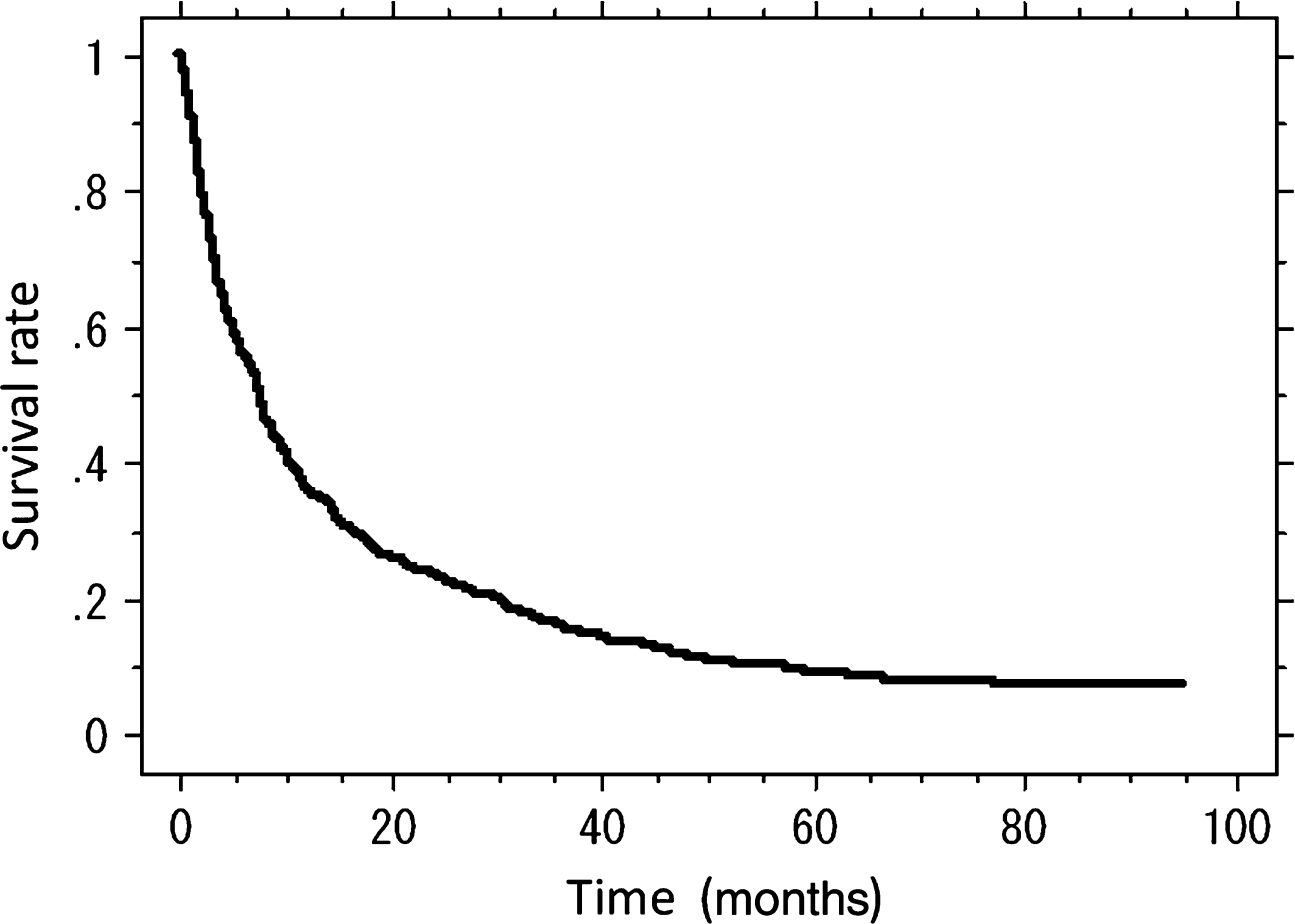
Kaplan–Meier cumulative survival rate for the 808 patients.

The overall rate of survival of the entire group was 0.57 at 6 months, 0.36 at 12 months, 0.23 at 24 months, and 0.16 at 36 months (Fig.1). Using multivariate analysis, the primary tumor group, visceral or cerebral metastases, abnormality of laboratory data, ECOG PS 3 or 4 grade, previous chemotherapy, and multiple skeletal metastases were found to be significantly independent prognostic factors (Table 3).

**Table 3:** Multivariate analysis of prognostic factors

Variable (coding)	Hazard ratio	(95% confidence interval)		*P*-value
Patient-related factor				
Gender (female: 0, male: 1)	1.14	0.97	1.35	0.113
Age in years (≤64: 0; ≥65: 1)	1.08	0.92	1.27	0.336
ECOG PS (PS 0–2: 0; PS 3–4: 1)	2.23	1.83	2.71	<0.001
Neurological deficits (Frankel D, E: 0; Frankel A–C: 1)	0.77	0.52	1.12	0.173
Laboratory data				
Normal (reference group)				
Abnormal	1.93	1.57	2.38	<0.001
Critical	2.87	2.23	3.69	<0.001
Primary-site-related factor				
Primary site				
Slow growth (reference group)				
Moderate growth	2.63	1.98	3.50	<0.001
Rapid growth	5.09	3.82	6.78	<0.001
Visceral or brain metastasis				
No metastasis (reference group)				
Nodular metastasis	1.89	1.46	2.44	<0.001
Disseminated metastasis	3.06	2.32	4.04	<0.001
Remaining primary lesion^1^	0.93	0.78	1.10	0.394
Previous chemotherapy^1^	1.39	1.18	1.65	<0.001
Skeletal-metastasis-related factor				
Location of skeletal metastasis				
Axial bone only (reference group)				
Axial bone and proximal limb	1.09	0.92	1.29	0.347
Spreading to distal limb	1.39	0.89	2.16	0.148
Multiple skeletal metastases^1^	1.55	1.29	1.88	<0.001
Pathological fracture^1^	0.98	0.83	1.17	0.839

1 Absent: 0; present: 1.

The highest hazard ratios were found for the primary tumor, with hazard ratios of 5.09 for the rapid growth group and 2.63 for the moderate growth group. The results indicated that patients with a rapid growth group primary site and those with a moderate growth group primary site were 5.09 and 2.63 times, respectively, more likely to die than those with a slow growth group primary site. The hazard ratios for ordinary visceral or cerebral metastases and disseminated metastases were 1.89 and 3.06, respectively. The hazard ratios for abnormal laboratory data and critical laboratory data were 1.93 and 2.87, respectively. The hazard ratios for three other significant factors, ECOG PS 3 or 4, previous chemotherapy, and multiple skeletal metastases were 2.23, 1.39, and 1.55, respectively (Table 3).

### Scoring system

**Figure 2:**
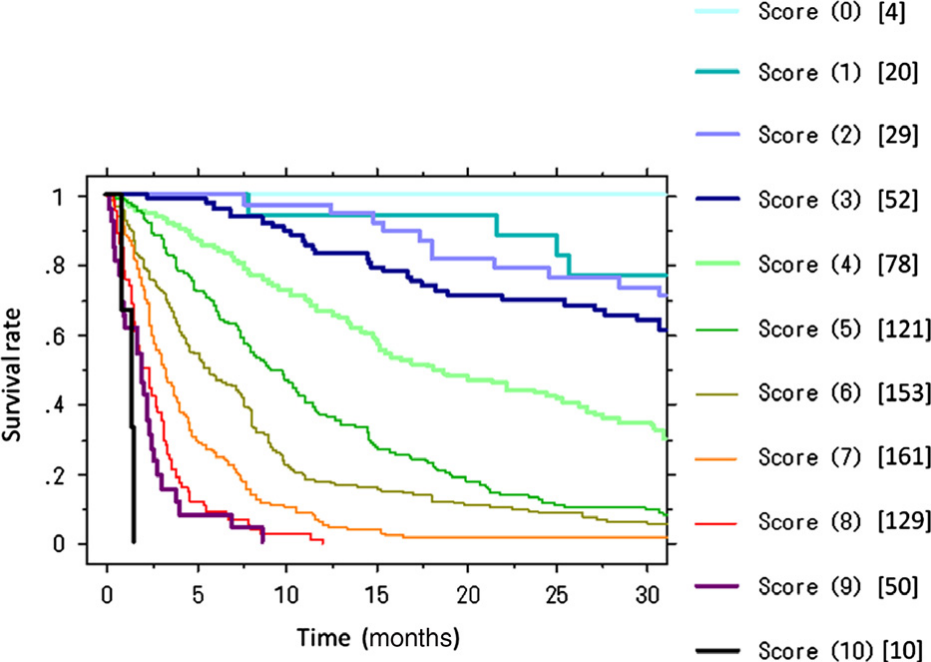
Kaplan–Meier survival curves for different prognostic scores. Values in parentheses and brackets indicate the prognostic scores and the number of patients, respectively. Survival rates deteriorate with increase in the prognostic scores.

The score for each significant prognostic factor was derived from the corresponding estimated regression coefficients (natural logarithm of the hazard ratio). The corresponding estimated regression coefficients were multiplied by 2 and rounded off to the nearest integer. This made the calculation of the prognostic score as simple as possible by allocating one point for factors with the smallest regression coefficients. As for the primary site, the rapid growth group was given three points and the moderate growth group was given two. Disseminated metastasis was given two points and ordinary metastasis was given one. Critical data were given two points and abnormal data were given one. Poor PS, previous chemotherapy, and multiple bone metastases were each given one point (Table 4). The prognostic score was calculated by adding together all of the scores for individual factors. Every patient was scored from 0 to 10, divided into 11 groups according to the prognostic score, and survival rates for each group were calculated. The Kaplan–Meier survival curves for different prognostic scores clearly demonstrated that the higher the prognostic score was, the lower the survival rate became (Fig.2, Table 5). For example, a patient with breast cancer without hormone receptors (2 points) who had ordinary visceral metastasis (1 point) and elevated CRP (1 point), with poor PS (1 point), and multiple bone metastasis development (1 point) after receiving chemotherapy (1 point) would have a total score of seven points (2 + 1 +1 + 1 + 1 + 1 = 7). This score was associated with a 1-year survival rate of 10% (Table 5).

**Table 4:** Significant prognostic factors and score for each factor

Prognostic factor		Regression coefficient	Score
**Primary site**			
Slow growth	Hormone-dependent breast and prostate cancer, thyroid cancer, multiple myeloma, and malignant lymphoma		0
Moderategrowth	Lung cancer treated with molecularly targeted drugs, hormone-independent breast and prostate cancer, renal cell carcinoma, endometrial and ovarian cancer, sarcoma, and others	0.99	2
Rapid growth	Lung cancer without molecularly targeted drugs, colorectal cancer, gastric cancer, pancreatic cancer, head and neck cancer, esophageal cancer, other urological cancers, melanoma, hepatocellular carcinoma, gall bladder cancer, cervical cancer, and cancers of unknown origin	1.70	3
**Visceral metastasis**	Nodular visceral or cerebral metastasis	0.65	1
	Disseminated metastasis^1^	1.11	2
**Laboratory data**	Abnormal^2^	0.64	1
	Critical^3^	1.04	2
**ECOG PS**	3 or 4	0.73	1
**Previous chemotherapy**		0.32	1
**Multiple skeletal metastases**		0.43	1
Total			10

1 Disseminated metastasis: Pleural, peritoneal, or leptomeningeal dissemination.

2 Abnormal: CRP ≥ 0.4 mg/dL, LDH ≥ 250 IU/L, or serum albumin <3.7 g/dL.

3 Critical: platelet <100,000/*μ*L, serum calcium ≥10.3 mg/dL, or total bilirubin ≥1.4.

**Table 5:** Prognostic scores and survival rates at 6, 12, and 24 months

Prognostic score	Number of patients	Survival rate		
		6 months	12 months	24 months
0	4	1.0	1.0	1.0
1	20	1.0	0.95	0.85
2	29	1.0	0.97	0.83
3	52	0.96	0.87	0.71
4	78	0.95	0.75	0.53
5	121	0.78	0.53	0.31
6	153	0.60	0.33	0.12
7	161	0.40	0.10	0.04
8	129	0.21	0.04	0.01
9	51	0.08	0	0
10	10	0	0	0

### Outcome

**Figure 3:**
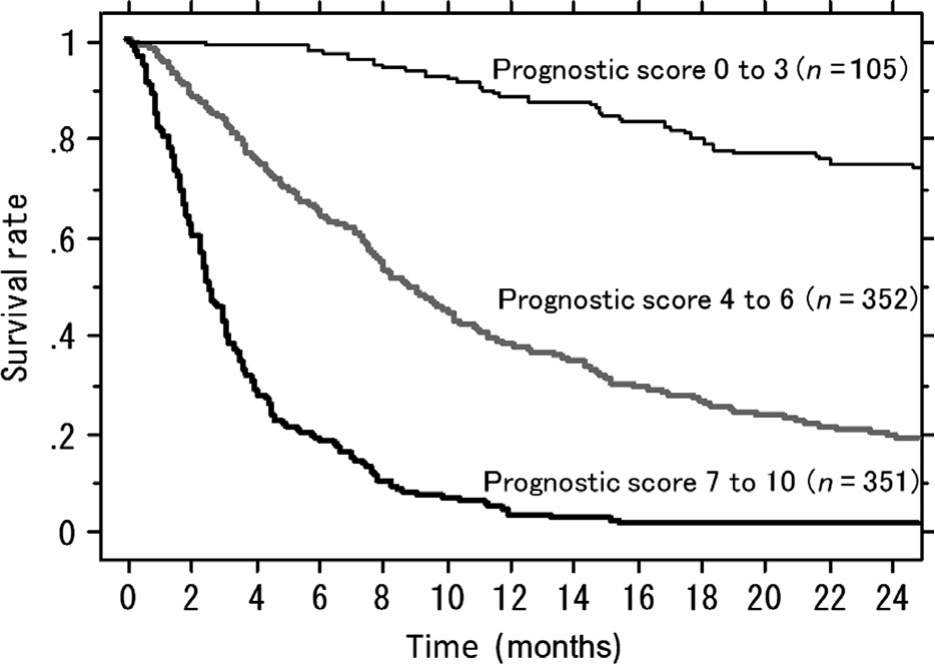
Kaplan–Meier survival curves for patients with prognostic scores of 0–3 (low-risk group), 4–6 (intermediate-risk group), and 7–10 (high-risk group). The rates of survival for these three groups are significantly different.

Judging from the survival rate at 12 months, the survival curves could be separated into three groups: a score of ≤3 for a survival rate of >80% at 12 months (low-risk group: 13% of the total population); a score of 4–6 for a survival rate of 30–80% (intermediate-risk group: 44% of the total population), and a score of 7–10 for a survival rate of ≤10% (high-risk group: 43% of the total population). The survival rates for these three groups were significantly different (log-rank test, *P* < 0.0001; Figure3; Table 6).

**Table 6:** Prognostic score and survival rate at 6, 12, and 24 months after detection of bone metastasis

Prognostic score	Survival rate (95% confidence interval)		
	6 months	12 months	24 months
0–3	0.981 (0.956–1.000)	0.914 (0.859–0.969)	0.778 (0.698–0.858)
4–6	0.740 (0.693–0.787)	0.493 (0.440–0.546)	0.276 (0.229–0.323)
7–10	0.269 (0.222–0.316)	0.060 (0.035–0.085)	0.021 (0.005–0.037)

### Comparison of the previous and the current system

We also analyzed the cohort of this study using a previously developed prognostic system [11]. To verify the improvement offered by our new system, we analyzed the patients whose risk classification differed between the two systems. As for the low-risk group, survival periods >1 year are expected. Among 60 patients who were classified as low risk using the current system but as intermediate risk using the previous system, seven patients (12%) died within 12 months and their risk status was wrongly predicted. Conversely, among 23 patients who were classified as low risk using the previous system but as intermediate risk using the current system, seven of 20 (35%) died within 12 months and were wrongly predicted. This difference was significant (Fisher's exact test, *P* < 0.001; Table 7). In the high-risk group, survival was expected to be <12 months. Among 65 patients who were categorized as high risk in the current system but as intermediate risk in previous system, only six (9%) survived >12 months and were wrongly predicted. While in the 96 patients who were high risk in previous system but intermediate in the current one, as many as 46% survived >12 months. This difference was also significant (Table 7). These results indicate that the current system can be used to stratify the patients more precisely than the previous system.

**Table 7:** Comparison between the new scoring system and the previous scoring system by analyzing patients differently classified using the two systems

Discrepancy pattern	Number of patients	12 months		24 months	
		Dead	Surviving	Dead	Surviving
Low-risk group					
Low risk in new system but intermediate risk in previous system	60	7	53	16^1^	41
Low risk in previous system but intermediate risk in new system	23	7^2^	13	12	8
Fisher's exact test		*P* < 0.001		*P* = 0.001	
High-risk group					
High risk in new system but intermediate risk in previous system	65	59	6	62	3
High risk in previous system but intermediate risk in new system	96	46^3^	44	67^4^	22
Fisher's exact test		*P* < 0.001		*P* = 0.001	

1 Three patients were censored within 24 months.

2 Three patients were censored within 12 months.

3 Six patients were censored within 12 months.

4 Seven patients were censored within 24 months.

## Discussion

There have been three prominent studies by orthopedic surgeons regarding prognostic factors for patients with skeletal metastasis 6–8. Studies by Tomita et al. 6. and Bauer et al. 8. involved the analysis of patients who had undergone surgery. The study by Tokuhashi et al. 7., analyzed both surgically and conservatively treated patients; two-thirds of the patients in their study underwent surgery. However, in a recent prospective study, the number of surgeries performed was approximately 7% of that of radiotherapies 18. Therefore, patients enrolled in these three studies might not be representative of patients with bone metastasis.

There have been similar large studies conducted by radiation oncologists 3,9,10. Although the majority of the patients with bone metastasis are treated by radiation therapy, the studies also appeared to have selection bias because they excluded surgically treated patients. In addition, Rades et al. 3. selected patients who had spinal cord compression and van der Linden et al. 9. only selected patients without any evidence of significant neurologic involvement, or without collapse or instability of the spine. To minimize the selection bias, we registered patients prospectively, including all those who had newly developed skeletal metastasis regardless of the treatments they had received.

Primary tumor and visceral or brain metastases have been the most commonly used prognostic factors in past studies 3,6–11, followed by poor PS 7,9–11. Above all, the primary tumor is considered to have the greatest impact on survival. However, a classification system for primary tumors seems not to have been established. Some previous studies have classified the primary tumor empirically without a firm statistical background 6,10,11. In contrast, primary tumors have been classified according to statistical analysis in some studies 3,8,9, but their classifications put too much emphasis on the common primary cancers. Consequently, malignancies other than breast, prostate, and lung cancer (which account for approximately 50% of primary lesions) are included in a single category termed “others” or no attention has been paid to them. Conventionally, lung cancer is considered to have the worst prognosis, although recent progress with chemotherapy using molecularly targeted drugs has enabled some patients to survive much longer than before. Currently, responses to gefitinib and erlotinib have become predictable by using gene analysis 19. In addition, sensitivity to hormonal therapy has also had a significant positive impact on the survival of patients with breast or prostate carcinoma 20,21. In this study, we took these concepts into account and categorized the primary tumors into three groups based on survival analysis for every primary site.

Laboratory data are known to be prognostic factors for some malignancies. However, laboratory data have not been sufficiently investigated as a prognostic factor in the past, with the exception of a study by Mizumoto et al. 10., which included serum calcium level. Our study suggested that laboratory data can be a significant prognostic factor.

Because bone metastases frequently occur in the spinal column, some studies have included neurological deficits as prognostic factors 3,7. Conversely, other studies have not considered neurological deficits as significant factors 6,8–11. We found no evidence showing that neurological deficit was a significant prognostic factor. The findings of the current analysis that poor PS, multiple bone metastasis, and previous chemotherapy are significant independent prognostic factors, but that age, gender, condition of the primary lesion, pathological fracture, and location of skeletal metastasis are not, are in accordance with a previous study by Katagiri et al. 11.

Some studies have attempted to establish a prognostic scoring system that designates patients either as those with spinal metastasis 3,6,7,9,10 or those with specific primary cancers 22,23. While it is true that the spine is the most common site for bone metastasis, skeletal metastasis is normally considered a systemic disease. Moreover, it is very common for patients with spinal metastasis to simultaneously have metastasis in other sites such as the pelvis or proximal extremities. Although neurological deficit is the only factor that is particular to spinal metastasis, it is not considered to be significant in many studies 6,8–11. A scoring system for a specific cancer could be precise, but would have the disadvantage of not being used in clinical practice. This is because doctors prefer to use a simple general system rather than multiple systems related to the primary lesion. Therefore, it is deemed better to establish a scoring system applicable to any symptomatic skeletal metastases by analyzing the patient as a whole, irrespective of the location of bone metastasis or primary lesion.

The characteristics of patients with bone metastasis are diverse. Therefore, it is sometimes difficult to choose from among the various types of radiotherapy and surgery. For patients with a prognostic score of ≤3, the expected rate of survival at 1 year is 91%. Therefore, long-course radiotherapy would be recommended because the rate of in-field recurrence is higher for short-course radiotherapy 2. If surgery is required, an excisional procedure followed by reconstruction is preferred, either for spinal metastasis or extremity metastasis because of long-lasting local control 4–7. In contrast, in patients with a prognostic score of ≥7, the expected rate of survival at 6 months is 27%, and only 6% at 1 year. This group of patients should probably be treated less invasively. If surgery is required, simple internal fixation is the first choice for patients with pathological fracture of the extremities. These patients are not good candidates for spinal surgery; therefore, radiotherapy with supportive care is the treatment of choice for patients with spinal metastases. For patients with a prognostic score of 4–6, the expected rate of survival is 49% at 1 year. If surgery is required, an excisional procedure followed by reconstruction, or internal fixation with augmentation by methylmethacrylate, is preferred for metastases of the lower limbs; and posterior instrumentation procedures would be preferred in the spine if radiotherapy is not expected to be effective.

There were some limitations regarding our study. First, the number of patients with some malignancies such as cervical cancer was small; only nine patients were enrolled. This is a consequence of the rarity of these cancers presenting with symptomatic bone metastasis. Another limitation was that only a small number of patients (7%) underwent surgery in this series; therefore, it might be difficult to draw a strong conclusion regarding surgical strategy. This may be partly as a consequence of the recent use of bisphosphonates which decreased skeletal-related events. To adequately address these issues and validate this study, a further prospective study involving the analysis of a population large enough to include a sufficient number of patients with cancers that infrequently develop skeletal metastasis, as well as a sufficient number of patients who underwent surgery, will be necessary.

When deciding upon treatment, not only the pain and degree of neurological impairment but also sensitivity to chemotherapy and/or radiotherapy, the destructive spread of bone metastases, and life expectancy must be considered. With this practical updated prognostic scoring system, life expectancy may be predicted more accurately; thus, a more optimal treatment may be selected.

## Conflict of Interest

None declared.

## References

[1] van der Linden YM, Steenland E, van Houwelingen HC, Post WJ, Oei B, Marijnen CA (2006). Dutch Bone Metastasis Study Group: patients with a favourable prognosis are equally palliated with single and multiple fraction radiotherapy: results on survival in the Dutch Bone Metastasis Study. Radiother. Oncol.

[2] Rades D, Stalpers LJ, Veninga T, Schulte R, Hoskin PJ, Obralic N (2005). Evaluation of 5 radiation schedules and prognostic factors for metastatic spinal cord compression. J. Clin. Oncol.

[3] Rades D, Fehlauer F, Schulte R, Veninga T, Stalpers LJ, Basic H (2006). Prognostic factors for local control and survival after radiotherapy of metastatic spinal cord compression. J. Clin. Oncol.

[4] Rompe JD, Eysel P, Hopf C, Heine J (1994). Metastatic instability at the proximal end of the femur: comparison of endoprosthetic replacement and plate osteosynthesis. Arch. Orthop. Trauma Surg.

[5] Wedin R, Bauer HC (2005). Surgical treatment of skeletal metastatic lesions of the proximal femur: endoprosthesis or reconstruction nail?. J. Bone Joint Surg. Br.

[6] Tomita K, Kawahara N, Kobayashi T, Yoshida A, Murakami H, Akamaru T (2001). Surgical strategy for spinal metastases. Spine.

[7] Tokuhashi Y, Matsuzaki H, Oda H, Oshima M, Ryu J (2005). A revised scoring system for preoperative evaluation of metastatic spine tumor prognosis. Spine.

[8] Bauer HCF, Wedin R (1995). Survival after surgery for spinal and extremity metastases. Acta Orthop. Scand.

[9] van der Linden YM, Dijkstra SP, Vonk EJ, Marijnen CA, Leer JW (2005). Prediction of survival in patients with metastases in the spinal column: results based on a randomized trial of radiotherapy. Cancer.

[10] Mizumoto M, Harada H, Asakura H, Hashimoto T, Furutani K, Hashii H (2008). Prognostic factors and a scoring system for survival after radiotherapy for metastases to the spinal column: a review of 544 patients at Shizuoka Cancer Center Hospital. Cancer.

[11] Katagiri H, Takahashi M, Wakai K, Sugiura H, Kataoka T, Nakanishi K (2005). Prognostic factors and a scoring system for patients with skeletal metastasis. J. Bone Joint Surg. Br.

[12] Harrington KD (1988). Orthopaedic management of metastatic bone disease.

[13] Frankel HL, Hancock DO, Hyslop G, Melzak J, Michaelis LS, Ungar GH (1969). The value of postural reduction in the initial management of closed injuries of the spine with paraplegia and tetraplegia. I. Paraplegia.

[14] Atzpodien J, Royston P, Wandert T, Reitz M (2003). Metastatic renal carcinoma comprehensive prognostic system. Br. J. Cancer.

[15] Han Y, Mao F, Wu Y, Fu X, Zhu X, Zhou S (2011). Prognostic role of C-reactive protein in breast cancer: a systematic review and meta-analysis. Int. J. Biol. Markers.

[16] Pugh RN, Murray-Lyon IM, Dawson JL, Pietroni MC, Williams R (1973). Transection of the esophagus for bleeding esophageal varices. Br. J. Surg.

[17] Kratz A, Pesce MA, Fink DJ, Fauci AS, Braunwald E, Kasper DL, Hauser SL, Longo DL, Jameson JL, Loscalzo J (2008). Appendix: laboratory values of clinical importance. Harrison's principles of internal medicine.

[18] Lipton A, Fizazi K, Stopeck AT, Henry DH, Brown JE, Yardley DA (2012). Superiority of denosumab to zoledronic acid for prevention of skeletal-related events: a combined analysis of 3 pivotal, randomised, phase 3 trials. Eur. J. Cancer.

[19] Lynch TJ, Bell DW, Sordella R, Gurubhagavatula S, Okimoto RA, Brannigan BW (2004). Activating mutations in the epidermal growth factor receptor underlying responsiveness of non-small-cell lung cancer to gefitinib. N. Engl. J. Med.

[20] Payne SJL, Bowen RL, Jones JL, Wells CA (2008). Predictive markers in breast cancer: the present. Histopathology.

[21] Petrylak DP (2002). Chemotherapy for androgen independent prostate cancer. Semin. Urol. Oncol.

[22] Sugiura H, Yamada K, Sugiura T, Hida T, Mitsudomi T (2008). Predictors of survival in patients with bone metastasis of lung cancer. Clin. Orthop. Relat. Res.

[23] Rades D, Douglas S, Huttenlocher S, Veninga T, Bajrovic A, Rudat V (2012). Prognostic factors and a survival score for patients with metastatic spinal cord compression from colorectal cancer. Strahlenther. Onkol.

